# *Chlamydia pneumoniae* facilitates its development by recruiting PI4P to inclusion bodies via the Cpn0308-ACBD3-PI4KB pathway

**DOI:** 10.1128/iai.00580-25

**Published:** 2026-06-04

**Authors:** Xiaonan Feng, Tian’ai Cao, Xiaohui Jia, Zewei Jia, Ping Li, Yan Zhou, Tianjun Jia

**Affiliations:** 1 Key Laboratory of Clinical Laboratory Diagnostics, Institute of Pathogenic Biology and Immunology, Hebei North University 261761, Zhangjiakou, China; 2 The Second Affiliated Hospital of Hebei North University, Zhangjiakou, China; University of California Davis, Davis, California, USA

**Keywords:** *C. pneumoniae*, Cpn0308, ACBD3, PI4KB, PI4P, inclusion membrane protein

## Abstract

*Chlamydia pneumoniae* (Cpn) is an obligate intracellular parasitic pathogen that replicates inside a membrane-bound vacuole termed the inclusion. This compartment is modified by chlamydial inclusion membrane proteins (Incs), which interact with host proteins to facilitate chlamydial development and pathogenicity. Among these, Cpn0308 localizes specifically to the inclusion membrane. Our previous study demonstrated that exogenously expressed Cpn0308 can interact with host protein ACBD3; here, we demonstrate this interaction during actual Cpn infection using endogenous proteins. This interplay proved critical for Cpn development, as ACBD3 knockout in HeLa cells led to significantly smaller inclusions, delayed transition from elementary bodies (EBs) to reticulate bodies (RBs), and reduced bacterial copy numbers. Mechanistically, the Cpn0308-ACBD3 interaction facilitates the recruitment of Phosphatidylinositol 4-kinase 3β (PI4KB) to the inclusion vicinity. This recruitment enhances the production and localization of Phosphatidylinositol 4-phosphate (PI4P) within Cpn*-*infected HeLa cells. Conversely, PI4KB and PI4P levels were markedly diminished in Cpn-infected ACBD3-knockout cells, whereas their expression remained unchanged in uninfected knockout cells. Furthermore, pharmacological inhibition of PI4KB with PIK93 impaired PI4P synthesis and suppressed Cpn replication. In conclusion, we propose a mechanism wherein the Cpn inclusion membrane protein Cpn0308 engages host ACBD3 to recruit PI4KB around the inclusion, which subsequently catalyzes PI4P production, thereby promoting the development and progression of *C. pneumoniae* infection.

## INTRODUCTION

*Chlamydia pneumoniae* is a gram-negative, obligate intracellular parasitic pathogenic microorganism, a common cause of various diseases in humans, such as upper respiratory infections and pneumonia, and also has been correlated with chronic diseases such as atherosclerosis, endocarditis, and Alzheimer’s disease (1–5). Like all chlamydiae, *C. pneumoniae* also has a biphasic developmental cycle, alternating between the elementary body (EB) and reticulate body (RB). The EB is an extracellular infectious but metabolically inert form that enters the host cell via endocytosis. Once inside, it is confined to a vacuole known as an inclusion, where it rapidly transforms into a non-infectious, replicating, and metabolically active reticulate body (RB). After undergoing several rounds of replication, the progeny RBs differentiate back into EBs, ready to infect new host cells (6–10). During the chlamydial development cycle, the inclusion acts as a specialized compartment, providing a safe environment for chlamydial replication and growth (11, 12). To ensure successful intravacuolar development, *Chlamydia* evolved mechanisms to import nutrients and metabolic intermediates from host cells into these inclusions. Inclusion membrane proteins (Incs), encoded by chlamydial genes, are expressed on the inclusion membrane and secreted via the *Chlamydia*-specific type III secretion system (T3SS). These Incs are considered crucial for mediating interactions between *Chlamydia* and host cells (13–17).

Thus, extensive efforts have been dedicated to identifying Incs, understanding the interactions between different Incs and host proteins, and elucidating their corresponding functions. For instance, Cpn1027 was identified to interact with Caprin2 and GSK3β, playing a role in the Wnt signaling pathway to enhance chlamydial anti-apoptotic activity (18); Cpn0585 interacts with several Rab GTPases, contributing to the development of *C. pneumoniae* (19). Additionally, Cpn0147 was studied for its interaction with cAMP-responsive element (CRE)-binding protein 3 (CREB3) (20). While Cpn0308 was previously identified as an inclusion membrane protein, there have been limited studies conducted to explore the effects of this Inc (21). Our previous research revealed that Cpn0308, expressed from an exogenously transfected recombinant plasmid, interacts with ACBD3 in HeLa cells. This interaction was confirmed through immunoprecipitation assays and GST-pull down techniques (22). However, the specific role of ACBD3 in the development of *C. pneumoniae* remains to be elucidated.

ACBD3 is a Golgi resident protein, also known as PBR-associated protein 7 (PAP7), and Golgi complex-associated protein (GCP60). It plays a crucial role in maintaining the structure of the Golgi apparatus, mediating steroidogenesis, regulating iron homeostasis, and participating in neuronal differentiation and apoptosis. Additionally, ACBD3 has been implicated in tumorigenesis and other cellular processes (23–26). Several studies have revealed that multiple viruses, such as enteroviruses 71 and 68, kobuviruses, rhinovirus, and Aichi virus, utilize their 3A protein to interact with ACBD3, then recruit PI4KB to the RNA replication sites, facilitating viral replication. Furthermore, research has shown that enterovirus 71 infection induces the production of phosphatidylinositol-4-phosphate (PI4P) in ACBD3 and PI4KB-dependent manner (27, 28). PI4P is a type of lipid involved in signaling and vesicular transport to the plasma membrane (29–32). Moorhead et al. discovered that following chlamydial infection, multiple host proteins recruited to the chlamydial inclusion can promote the synthesis of PI4P and its presence at the inclusion membrane. However, the exact mechanism underlying this process remains unknown (33).

Since our group has discovered that exogenous Cpn0308 interacts with the host protein ACBD3, we further validated this interaction between the inclusion membrane protein Cpn0308 and ACBD3 through co-immunoprecipitation in *C. pneumoniae*-infected HeLa cells. Additionally, by utilizing CRISPR-Cas9 technology to generate ACBD3-knockout HeLa cells, we demonstrated the important role of ACBD3 in the development of *C. pneumoniae*. Our study also revealed that Cpn0308 utilizes ACBD3 to recruit PI4KB, thereby enhancing the synthesis of PI4P. This process plays a pivotal role in facilitating *C. pneumoniae* replication.

## RESULTS

### Cpn0308 was found to co-localize and interact with the host protein ACBD3 in HeLa cells infected with *C. pneumoniae*

**Fig 1:**
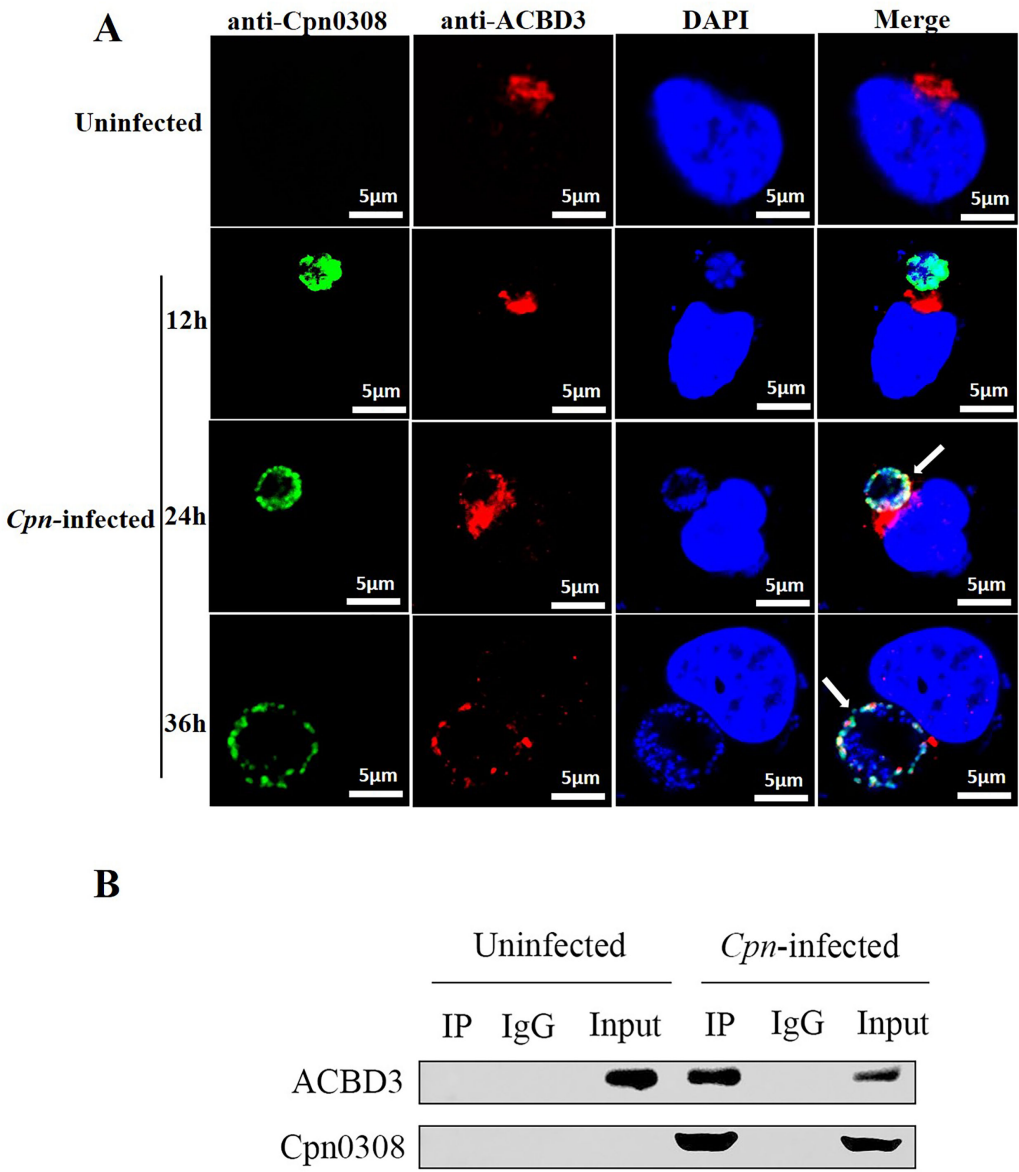
Co-localization and interaction of Cpn0308 with ACBD3 in *C. pneumoniae* infected HeLa cells. (**A**) Co-localization of Cpn0308 with ACBD3 was detected by immunofluorescence. HeLa cells were either infected (Cpn*-*infected) or uninfected with *C. pneumoniae*. At 12, 24, and 36 h post-infection, the cells were fixed and labeled with antibodies against Cpn0308 (green) and ACBD3 (red). DAPI staining (blue) indicates the locations of the cell nuclei. The images were acquired using a confocal laser scanning microscope. Scale bar = 5 μm. (**B**) The interaction of Cpn0308 with ACBD3 was detected by co-immunoprecipitation (Co-IP) assay. HeLa cells were infected with *C. pneumoniae* for 24 h. The cell lysates were prepared and immunoprecipitated with antibody against Cpn0308. Samples were analyzed by Western blotting using antibodies against Cpn0308 and ACBD3 (Input: the cell lysates were analyzed by Western blotting using antibodies against Cpn0308 and ACBD3; IgG: the cell lysates were immunoprecipitated with a non-specific antibody and analyzed by Western blotting using antibodies against Cpn0308 and ACBD3). The data shown are representative of the results of three independent experiments.

In our previous study, we observed that Cpn0308 co-localized and interacted with ACBD3 when plasmids pcDNA3.1(+)/Flag-ACBD3 and pcDNA3.1(+)/Myc-His-Cpn0308 were co-transfected into HeLa cells (22). In the present investigation, we infected HeLa cells with *C. pneumoniae* to further analyze the co-localization and interaction between Cpn0308 and the host protein ACBD3. The cells were subjected to indirect immunofluorescence labeling and examined using confocal microscopy at different time points after infection. As illustrated in Fig. 1A, *C*. *pneumoniae*-infected cells exhibited characteristic inclusion formation, confirmed by positive immunoreactivity against Cpn0308. Notably, while ACBD3 and Cpn0308 showed no overlapping localization at 12 h post-infection, progressive co-localization became increasingly apparent at 24 and 36 h during the infectious time course. In contrast, uninfected cells completely lacked inclusion structures, and correspondingly, no ACBD3-Cpn0308 co-localization was detectable under these conditions.

Since the observed co-localization of ACBD3 and Cpn0308 at 24 h post-infection, we chose to identify their interaction at this time using co-immunoprecipitation (Co-IP). Cell lysates from HeLa cells infected with *C. pneumoniae* for 24 h were prepared and analyzed by immunoblotting with an anti-Cpn0308 antibody. The results showed clear bands at molecular weights of 15 and 75 kDa, corresponding to Cpn0308 and ACBD3, respectively. No such bands were observed in the lysates of uninfected cells, indicating a direct interaction between Cpn0308 and ACBD3 (Fig. 1B).

### ACBD3 plays a pivotal role in the development of *C. pneumoniae*

**Fig 2:**
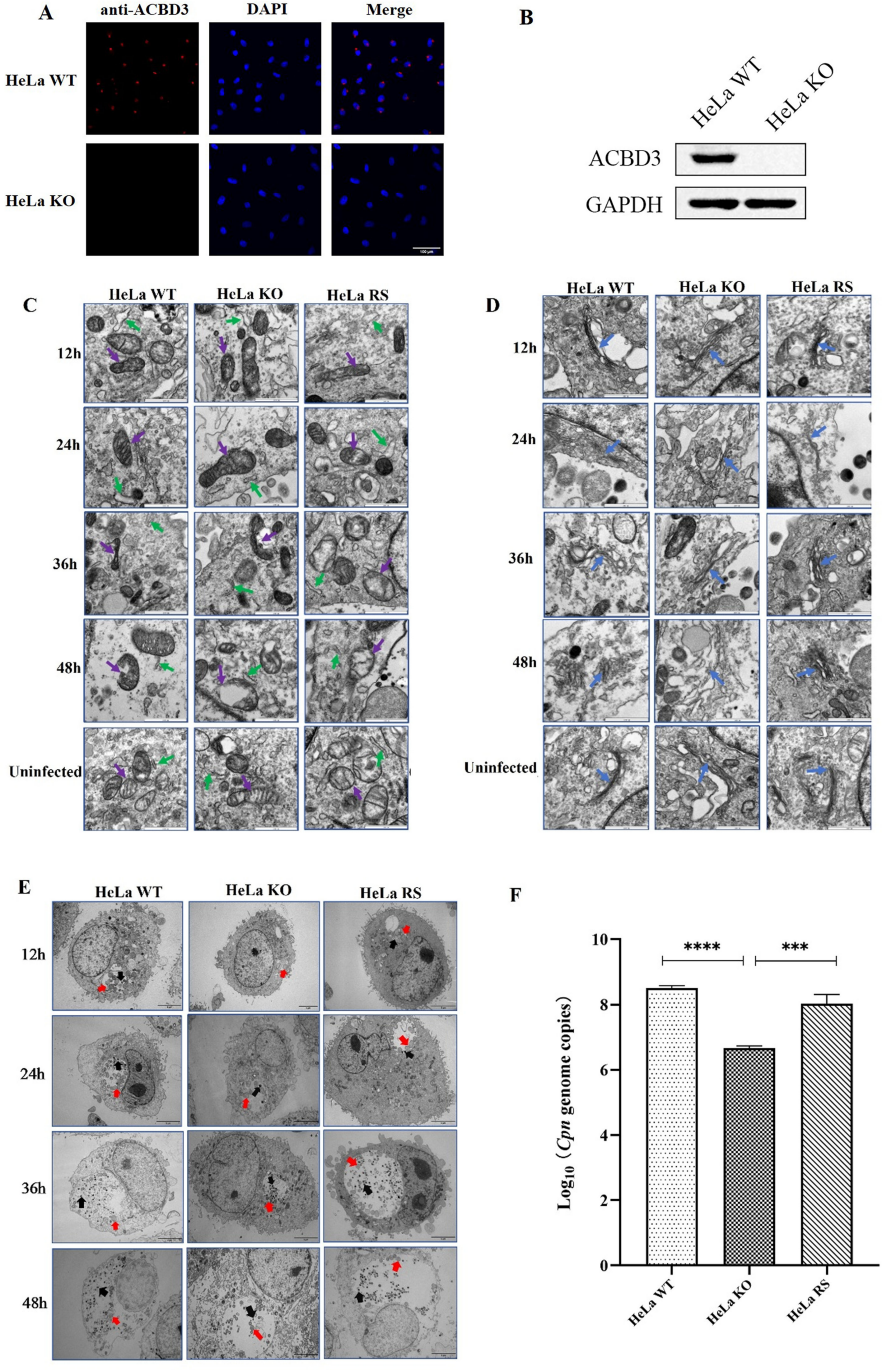
ACBD3 is vital for *C. pneumoniae* development. (**A and B**) CRISPR/Cas9 technology was employed to generate ACBD3-deficient HeLa cells. The absence of ACBD3 expression in these cells was confirmed via indirect immunofluorescence (**A**) and Western blotting (**B**), where cells were fixed in 4% paraformaldehyde and stained with anti-ACBD3 (red), and examined under the laser confocal microscopy (200×, scale bar = 25 µm). (**C–E**) HeLa KO, HeLa WT, and HeLa RS cells were infected with *C. pneumoniae* for 12, 24, 36, and 48 h. Morphological alterations in mitochondria (C, 15,000×), endoplasmic reticulum (**C**, 15,000×), and Golgi apparatus (**D**, 25,000×), as well as development of *C. pneumoniae* (**E**), were observed using transmission electron microscopy. Mitochondria are indicated by purple arrows, endoplasmic reticulum is indicated by green arrows, and Golgi are indicated by blue arrows; scale bar = 1 µm. Additionally, EBs are marked by black arrows, and reticulate bodies (RBs) by red arrows; scale bar = 5 µm. (**F**) The DNA levels of the inclusion membrane protein Cpn0308 were quantified using quantitative real-time PCR with TaqMan probes. HeLa KO, HeLa WT, and HeLa RS cells were infected with *C. pneumoniae* for 24 h, followed by total cellular DNA extraction using a Genomic DNA Extraction Kit. A recombinant plasmid PGBKT7-Cpn0308, prepared in our laboratory, served as the standard. Values were statistically evaluated using a one-way ANOVA. ***, *P*<0.001; ****, *P*<0.0001. The data shown are representative of the results of three independent experiments. HeLa KO: ACBD3 knockout HeLa cells; HeLa WT: wild-type HeLa cells; HeLa RS: ACBD3 restored HeLa cells (transfected with a plasmid expressing ACBD3 into HeLa KO cells).

Lei’s research demonstrated that the Golgi protein ACBD3 enhances Enterovirus 71 replication by interacting with its 3A protein. Notably, an ACBD3 knockout impedes the replication of enteroviruses A to D and rhinovirus species A and B (28). Building on the established interaction between ACBD3 and Cpn0308, we postulated that ACBD3 might be crucial for the growth and development of *C. pneumoniae*. To validate this hypothesis, we employed CRISPR/Cas9 technology to generate HeLa cells lacking ACBD3. The successful ablation of ACBD3 was confirmed through indirect immunofluorescence and Western blotting assays. As illustrated in Fig. 2A and B, the lack of ACBD3 expression in HeLa cells confirmed the successful establishment of ACBD3-deficient HeLa cells (designated as HeLa KO). Subsequently, we used *C. pneumoniae* to infect these HeLa KO cells, along with wild-type HeLa cells (designated as HeLa WT) and ACBD3-restored HeLa cells (designated as HeLa RS, which were transfected with a plasmid expressing ACBD3). The three phenotypes of cells grew similarly (see Fig. S1 at https://figshare.com/s/bb165ab60c31b8be86d7). The transmission electron microscopy results revealed mitochondrial cristae disruption, endoplasmic reticulum swelling, and fragmentation of the Golgi apparatus (Fig. 2C and D). Notably, qualitative morphological assessment suggested that in HeLa KO cells, *C. pneumoniae* inclusions appeared smaller and exhibited a lower relative abundance of EB-like particles and RB-like particles compared to HeLa WT and HeLa RS cells (Fig. 2E).

To quantify *C. pneumoniae* replication, we measured the DNA levels of the inclusion membrane protein Cpn0308 using quantitative real-time PCR with Taqman probes after a 24-h infection period. As shown in Fig. 2F, the replication of *C. pneumoniae* in HeLa KO cells was significantly reduced relative to that in HeLa WT and HeLa RS cells. Furthermore, the reintroduction of ACBD3 in HeLa KO cells (HeLa RS cells) restored *C. pneumoniae* replication. Collectively, these findings demonstrated that ACBD3 significantly contributes to the intracellular proliferation of *C. pneumoniae*.

### *C. pneumoniae* promoted the co-localization and interaction of ACBD3 and PI4KB

**Fig 3:**
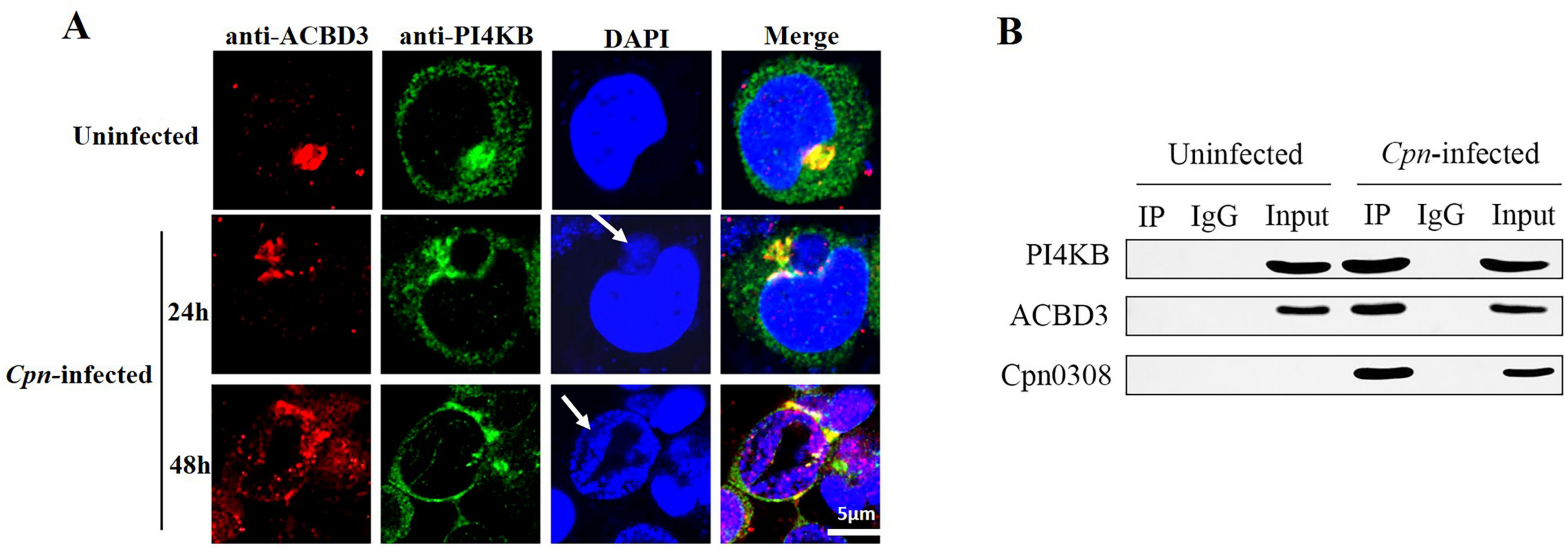
Confirmation of co-localization and interaction between ACBD3 and PI4KB in wild-type HeLa cells infected with *C. pneumoniae*. (**A**) Co-localization of ACBD3 with PI4KB was confirmed using immunofluorescence. HeLa cells were infected with *C. pneumoniae* for either 24 or 48 h or left uninfected as a control. The cells were then fixed and stained with antibodies against PI4KB (green) and ACBD3 (red). DAPI staining (blue) was used to indicate the locations of the cell nuclei. Images were captured using a confocal laser scanning microscope. The scale bar in each image corresponds to 5 µm. White arrows indicate the inclusion bodies. (**B**) The interaction between ACBD3 and PI4KB, as well as the interaction between Cpn0308 and PI4KB, was detected by co-immunoprecipitation (Co-IP). HeLa cells were infected with *C. pneumoniae* for 24 h, lysed, and then immunoprecipitated with an antibody against Cpn0308. Then, the samples were analyzed by Western blotting using antibodies against Cpn0308, ACBD3, and PI4KB. (Input: the cell lysates were analyzed by Western blotting using antibodies against Cpn0308, ACBD3, and PI4KB; IgG: the cell lysates were immunoprecipitated with non-specific antibody and analyzed by Western blotting using antibodies against Cpn0308, ACBD3, and PI4KB). The displayed data represent the results from three independent experiments.

Given the significant role of ACBD3 in promoting *Chlamydia* development, we aimed to elucidate the specific pathway influencing chlamydial progression. Ishikawa-Sasaki and Xiao found that viruses such as Aichi, Enterovirus 71, and Enterovirus 68 utilize their viral proteins to recruit PI4KB by interacting with ACBD3 at replication sites, thereby facilitating genome replication (29, 34). To explore whether *C. pneumoniae* could promote the co-localization of ACBD3 and PI4KB around inclusion bodies, we infected wild-type HeLa cells with *C. pneumoniae* for 24 and 48 h, stained with anti-ACBD3 and anti-PI4KB using indirect immunofluorescence assay, and observed under confocal microscopy, in uninfected HeLa cells, PI4KB partially co-localized with ACBD3. While at 24 h after infection, co-localization of PI4KB and ACBD3 was observed and aggregated near the inclusion body. At 48 h post-infection, a substantial fraction of ACBD3 and PI4KB were found to co-localize around the inclusion body (Fig. 3A). Next, we conducted co-immunoprecipitation (Co-IP) experiments to investigate the interaction between ACBD3 and PI4KB, as well as between Cpn0308 and PI4KB. After a 24-h period of infection with *C. pneumoniae*, lysate from HeLa cells was prepared and subsequently analyzed via immunoblotting using an antibody specific to Cpn0308. The results revealed distinct bands at molecular weights of 15, 70, and 110 kDa, corresponding to Cpn0308, ACBD3, and PI4KB, respectively. Notably, no such bands were observed in the lysates of uninfected cells (Fig. 3B). These findings indicated that *C. pneumoniae* promoted the co-localization and interaction of ACBD3 and PI4KB around inclusion bodies.

### ACBD3 is conducive to PI4KB production in *Chlamydia* infection

Previous studies have also demonstrated that ACBD3 is an essential Pan-enterovirus host factor that mediates the interaction between the viral 3A protein and cellular protein PI4KB (29, 34–37). We then assessed the importance of ACBD3 in the recruitment of PI4KB during *C. pneumoniae* infection by examining the localization and expression of PI4KB in ACBD3 knock-out HeLa cells.

**Fig 4:**
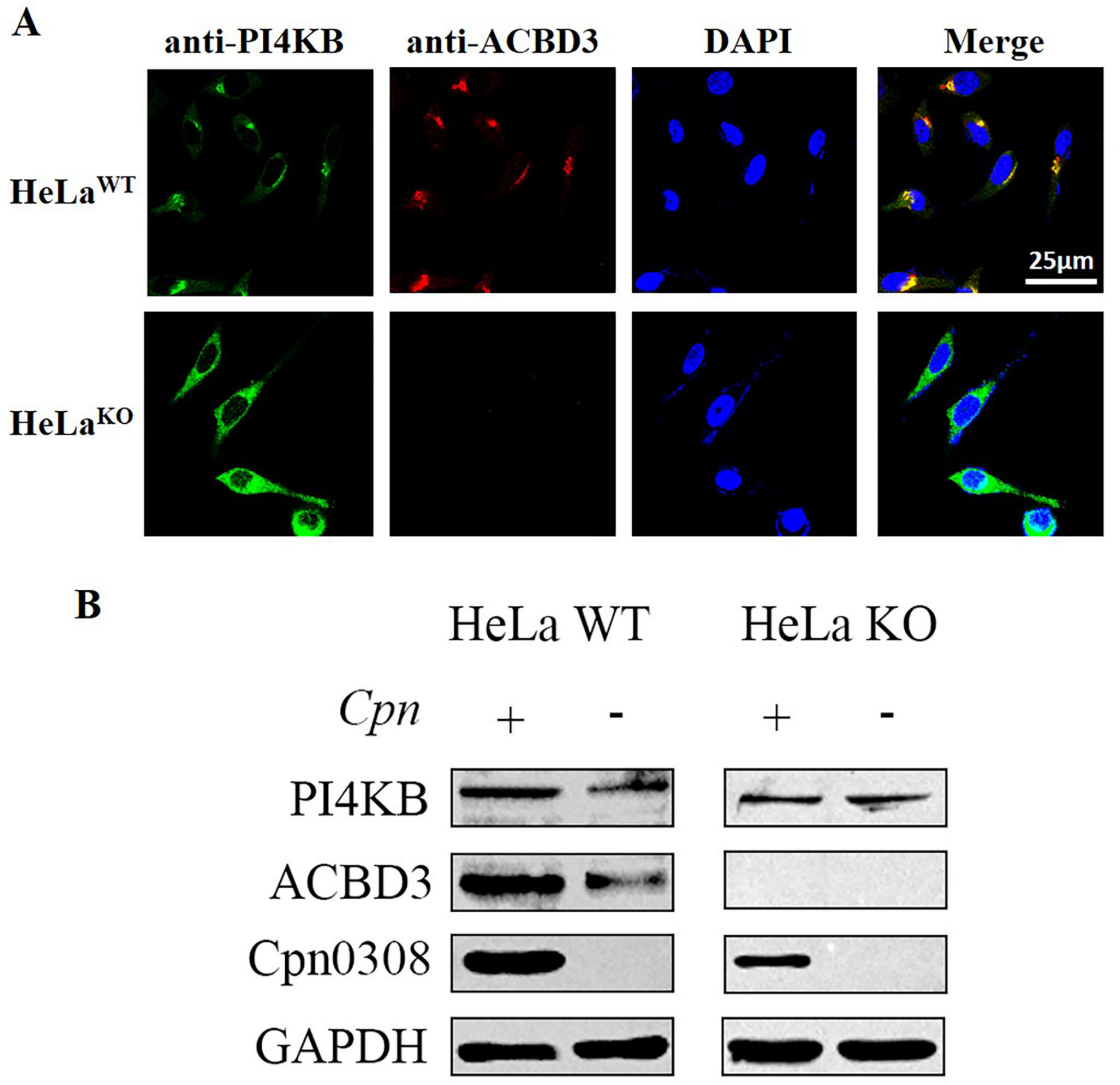
The localization and expression of PI4KB in ACBD3 knockout HeLa KO cells were detected using immunofluorescence and Western blotting analysis. (**A**) The cells were fixed with 4% paraformaldehyde and stained with anti-ACBD3 (red) and anti-PI4KB (green) antibodies. DAPI staining (blue) indicates the locations of the cell nuclei. The images were acquired using a confocal laser scanning microscope at 200× magnification. The scale bar in each image corresponds to 25 µm. (**B**) HeLa WT and HeLa KO cells were infected with *C. pneumoniae*. Cell lysates were extracted at 24 h post-infection and analyzed by Western blot using antibodies against PI4KB, ACBD3, Cpn0308, and GAPDH. The displayed data represent the results from three independent experiments.

HeLa KO and HeLa WT cells were incubated for 24 h, followed by fixation and immunostaining with anti-PI4KB and anti-ACBD3. Confocal microscopy analysis revealed distinct localization patterns: in ACBD3-deficient HeLa KO cells, PI4KB exhibited diffuse cytoplasmic distribution, whereas HeLa WT cells (with ACBD3) displayed concentrated PI4KB signals that prominently co-localized with ACBD3 (Fig. 4A). Western blot analysis of lysates from *Chlamydia*-infected cells demonstrated contrasting responses: *C. pneumoniae* infection induced no significant alteration in PI4KB expression in ACBD3-knockout HeLa KO cells, while PI4KB expression markedly increased in infected HeLa WT cells (Fig. 4B). These findings indicated that ACBD3 facilitates PI4KB upregulation during *Chlamydia* infection.

Additionally, we observed that the expression of Cpn0308 in *C. pneumoniae*-infected HeLa KO cells was significantly lower than that in *C. pneumoniae-*infected HeLa WT cells, consistent with the results shown in Fig. 2F.

### Chlamydial infection promotes the localization of PI4P on inclusion membranes and increases its synthesis via ACBD3

**Fig 5:**
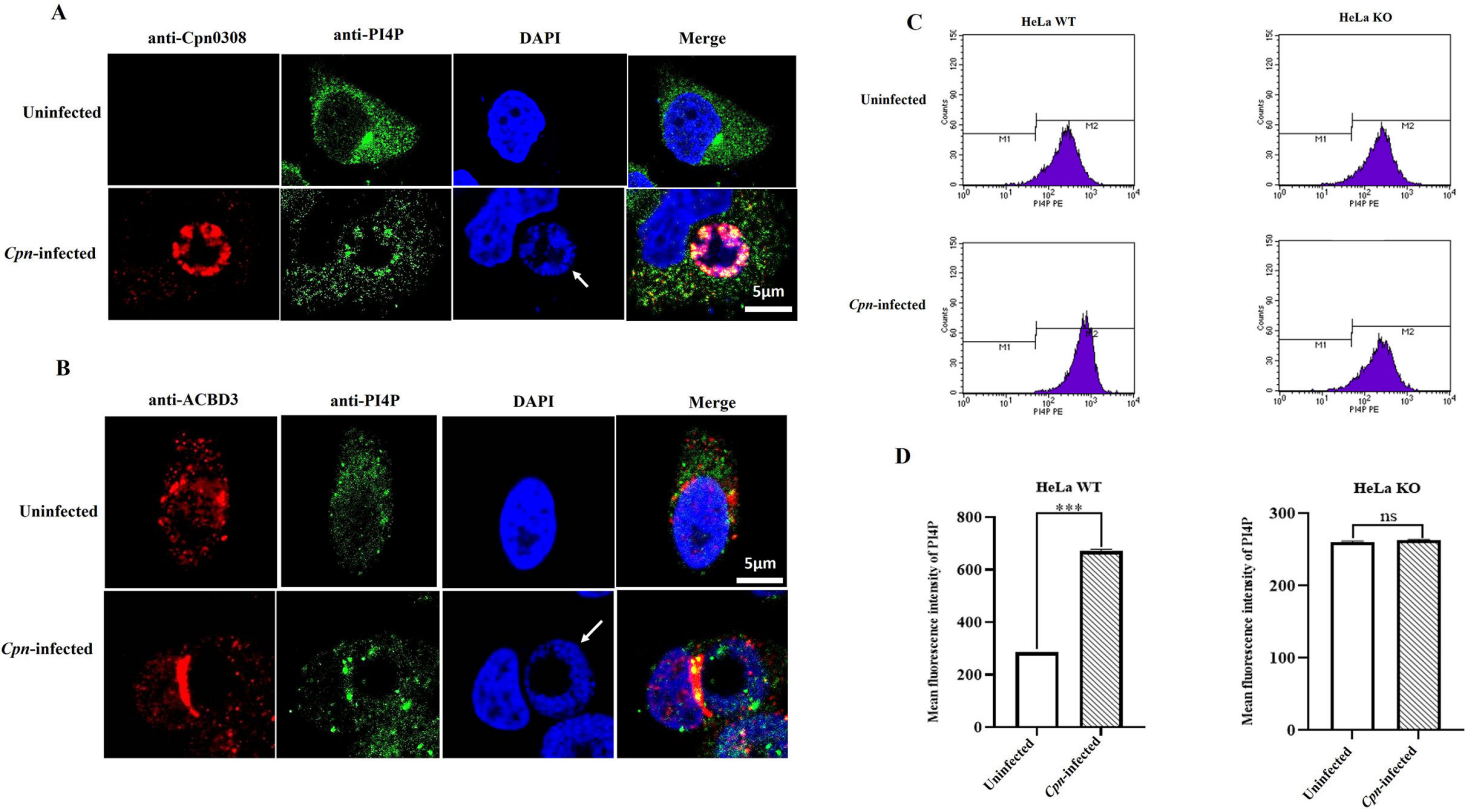
The localization and content of PI4P in HeLa WT and HeLa KO cells following *C. pneumoniae* infection. Co-localization of Cpn0308 with PI4P (**A**), ACBD3 with PI4P (**B**) was detected by immunofluorescence. HeLa WT cells were infected with *C. pneumoniae* and analyzed at 24 h. The cells were fixed with 4% paraformaldehyde and stained with primary antibodies against ACBD3 (red), Cpn0308 (red), and PI4P (green). DAPI staining (blue) indicates the locations of the cell nuclei. White arrows point to inclusion bodies. The images were acquired with a confocal laser scanning microscope. The scale bar in each image corresponds to 5 µm. (**C**) HeLa KO and HeLa WT cells were either infected with *C. pneumoniae* or not infected (uninfected). After 24 h, the cells were collected and fixed with 4% paraformaldehyde, labeled with anti-PI4P antibody, and then analyzed by flow cytometry. (**D**) Statistical analysis of data presented in panel (**C**) using one-way ANOVA (*n* = 3 independent experiments; ***, *P* < 0.001). HeLa KO: ACBD3 knockout HeLa cells; HeLa WT: wild-type HeLa cells. The data shown are representative of the results of three independent experiments.

Our findings indicate that ACBD3 promotes PI4KB upregulation and exhibits co-localization with it at the inclusion body periphery during *C. pneumoniae* infection. This suggests a potential role for ACBD3 in recruiting PI4KB to the pathogen’s replication site. Given that PI4KB represents a major phosphatidylinositol 4-kinase (PI4K) enzyme responsible for PI4P biosynthesis, we hypothesized that PI4P might be similarly recruited for synthesis and localization around inclusion bodies, participating in the development of *C. pneumoniae* infection. We then first analyzed the co-localization of ACBD3 and PI4P following *C. pneumoniae* infection. As illustrated in Fig. 5A, PI4P was diffusely distributed in the cytoplasm of uninfected cells but co-localized with Cpn0308 within inclusion bodies at 24 h post-infection. Similarly, Fig. 5B revealed ACBD3-PI4P co-localization specifically near inclusion membranes during infection, contrasting with cytoplasmic co-localization in uninfected cells. Integrating these findings with the Cpn0308-ACBD3 interaction confirmed in Fig. 1, we proposed that *C. pneumoniae* infection triggers Cpn0308-ACBD3-PI4KB engagement, subsequently recruiting PI4P to co-localize with them on inclusion membrane.

Next, we examined the changes in PI4P content 24 h following *C. pneumoniae* infection in HeLa KO and HeLa WT cells using flow cytometry to define whether *C. pneumoniae* affected PI4P via ACBD3. As illustrated in Fig. 5C and D, *C*. *pneumoniae* infection significantly increased the PI4P content in HeLa WT cells compared to the uninfected group. However, this upregulation of PI4P was excluded in HeLa KO cells. Therefore, the results indicated that *C. pneumoniae* promotes the synthesis of PI4P that is dependent on ACBD3. This is similar to the result illustrated in Fig. 4B that ACBD3 is in favor of PI4KB generation.

### PI4KB also plays a substantial role in PI4P production and *C. pneumoniae* replication

Xiao (29) discovered that Enterovirus 71 stimulates the synthesis of PI4P, which depends on both PI4KB and ACBD3. Since we found that PI4P production is dependent on ACBD3 and its interaction with PI4KB during *C. pneumoniae* infection, we then explored the effect of PI4KB on PI4P production and *C. pneumoniae* replication. To explore this effect, we treated HeLa cells with PIK93, a specific inhibitor of PI4KB.

**Fig 6:**
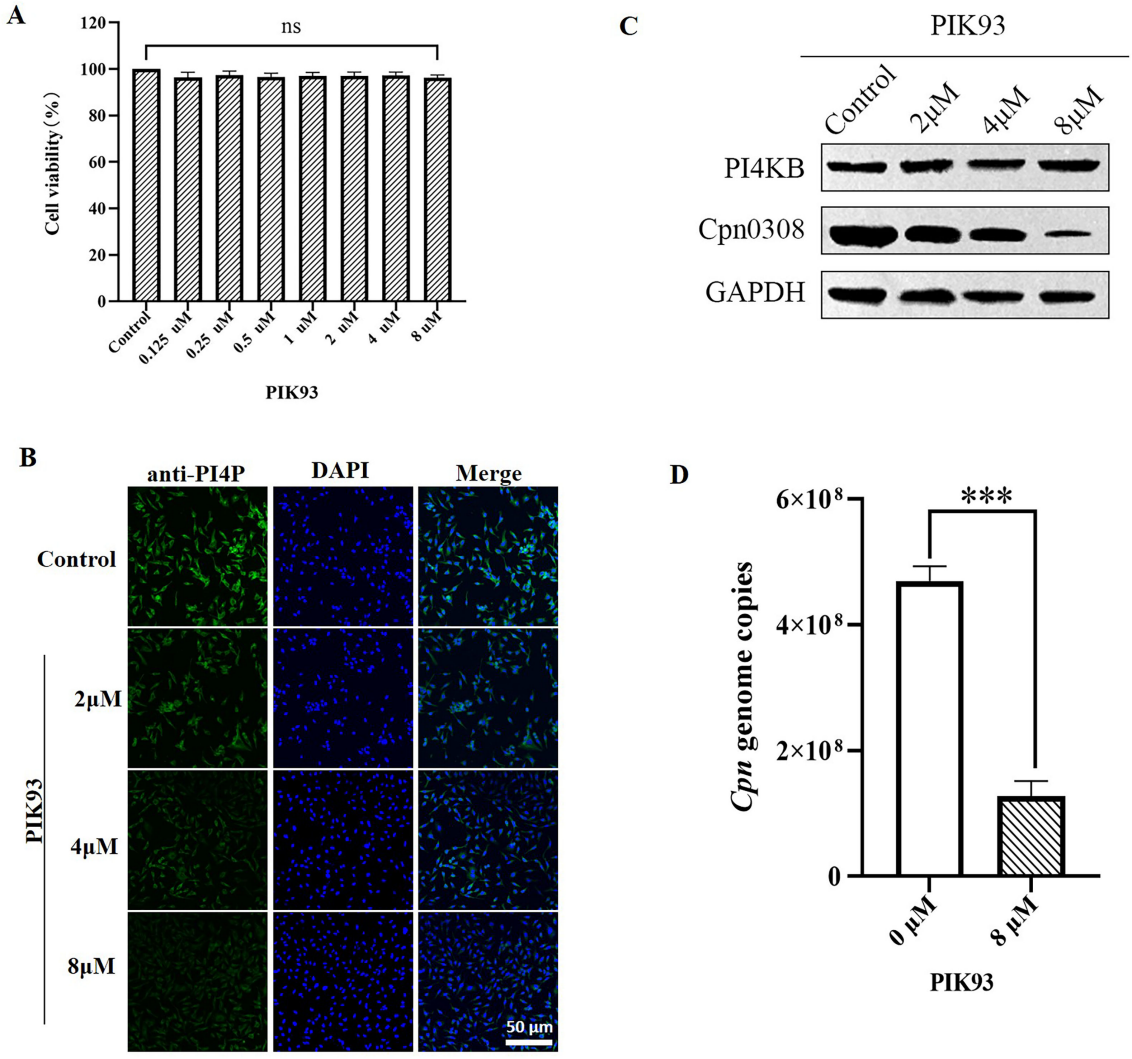
Effect of PI4KB on PI4P production and *C. pneumoniae* replication. HeLa WT cells were initially cultured for 24 h, after which they were exposed to varying concentrations of PIK93 for an additional 24 h. (**A**) The viability of the cells was evaluated using the MTT assay. (**B**) The expression levels of PI4P were observed and analyzed through the immunofluorescence. The images were acquired using a confocal laser scanning microscope at 100× magnification. The scale bar in each image corresponds to 50 µm. (**C**) HeLa WT cells treated with PIK93 were continuously infected with *C. pneumoniae* for 24 h, cell lysates were harvested and the expression of Cpn0308 and PI4KB was analyzed by Western blotting. (**D**) Total cellular DNA was extracted from HeLa WT cells treated with PIK93 and *C. pneumoniae* for 24 h, the impact of 8 μM PIK93 on the copy number of *C. pneumoniae* was assessed through real-time fluorescence quantitative PCR using Taqman probe. ***, *P*＜0.001. HeLa cells without treatment of PIK93 and *C. pneumoniae* were employed as controls.

We first screened suitable concentrations of PIK93 that were non-toxic to HeLa cells using an MTT assay. We found that there was no significant effect on cell viability when the drug concentration ranged from 0.125 to 8 µM (Fig. 6A). Subsequent treatment of HeLa cells with increasing concentrations of PIK93 (0, 2, 4, and 8 µM) revealed a dose-dependent decrease in PI4P fluorescence intensity (Fig. 6B). Western blot analysis revealed that PI4KB protein levels in HeLa cells remained largely unchanged, whereas the expression of the *C. pneumoniae* inclusion membrane protein Cpn0308 decreased progressively (Fig. 6C). Furthermore, *C. pneumoniae* replication was significantly inhibited at 8 µM PIK93, as quantified by TaqMan probe-based real-time PCR (Fig. 6D). This suppression of chlamydial growth may subsequently lead to reduced Cpn0308 expression. Together, these results indicate that PI4KB contributes critically to PI4P generation, Cpn0308 expression, and *C. pneumoniae* replication.

## DISCUSSION

*Chlamydia* establishes itself within host cells by forming inclusion bodies, where they complete their growth and development cycle. This process necessitates numerous host cellular mechanisms involving complex interactions between host cell molecules and chlamydial products (38). The chlamydial inclusion membrane serves as the critical interface between *Chlamydia* and the host cell. It undergoes extensive modification through the insertion of type III secreted inclusion membrane proteins (Incs), which are essential for maintaining the structure of the inclusion membrane and facilitating host-pathogen interactions (8, 38, 39). Our study was the first to identify an interaction between the *C. pneumoniae* inclusion membrane protein Cpn0308 and the host Golgi protein ACBD3 during *C. pneumoniae* infection. ACBD3 is a multi-functional scaffolding protein, playing unusually diverse roles both in normal cell physiology and in various diseases (40). To elucidate the role of the interaction between Cpn0308 and ACBD3 in *C. pneumoniae* development and its underlying mechanism, we generated ACBD3-knockout HeLa cells using the CRISPR-Cas9 technique.

In our initial observations, we noted that the mitochondrial structure in ACBD3-knockout HeLa cells remained largely unaffected in the absence of *C. pneumoniae* (Cpn) infection (Fig. 2C and D), which was also demonstrated by Daňhelovská et al.’s work (25), who reported that knockout of ACBD3 do not alter the cholesterol level and mitochondrial structure and functions are not altered, while ACBD3-knockout cells exhibit enlarged Golgi area with absence of stacks and ribbon-like formation, confirming the importance of ACBD3 in Golgi stacking. Our research also found that ACBD3-knockout HeLa cells exhibited Golgi fragmentation and a reduction in stack formation. These findings corroborate previously published data on ACBD3-downregulated and ACBD3-knockout HeLa cell lines which exhibit irregular ribbon and dramatic Golgi fragmentation (23–25). The observed effects may be attributed to ACBD3, a scaffolding protein of the Golgi apparatus, its interactions with several distinct proteins in different parts of Golgi function in range from maintenance of the Golgi structure, membrane trafficking, and glycosphingolipid metabolism to regulation of *de novo* fatty acid synthesis or apoptosis (36). Furthermore, our findings revealed an obvious Golgi apparatus fragmentation and a reduction in stack formation when these HeLa cells (ACBD3-knockout, wild-type, and ACBD3-restored) were infected with *C. pneumoniae*. This was accompanied by noticeable mitochondrial swelling and disruption of the cristae structure within the infected cells. These observations might be explained by previous studies of *Chlamydia trachomatis*. For example, Heuer’s study showed that infection of human epithelial cells by *Chlamydia trachomatis* induced similar Golgi fragmentation, then enhanced the transport of sphingolipids to the bacterial inclusion and facilitated efficient chlamydial growth (41). Other studies have also indicated that *Chlamydia trachomatis* remodels host cell mitochondria in various ways, including altering mitochondrial dynamics such as increasing mitochondrial fusion, promoting mitochondrial integrity, and interfering with the mitochondrial import machinery and protein composition. Thus, these modifications maintain a favorable environment for the reproduction and growth of *Chlamydia trachomatis* (42). Though there is no clear support that ACBD3 plays an independent function in mitochondria, and we did not specifically investigate the mitochondrial localization of ACBD3 in this study, we recognize the importance of this pathway and its potential impact on *Chlamydia* infection. Our future work will include a more detailed examination of mitochondrial dynamics and their interaction with ACBD3 during infection. Given *Chlamydia trachomatis and C. pneumoniae* belong to the same species, they have similar life cycles and interaction with their hosts to maintain their growth and development, *C. pneumoniae* might also sustain its growth and development by disrupting the Golgi apparatus and remodeling host cell mitochondria.

Furthermore, our morphological analysis showed that inclusions in ACBD3-knockout HeLa cells were comparatively smaller and enriched in EB-like forms (Fig. 2E). This may be interpreted as a potential alteration in developmental timing, which could include a slowed EB-to-RB differentiation or an imbalanced RB-to-EB conversion. Future quantitative morphometric work would be necessary to distinguish between these possibilities and to precisely characterize the observed developmental profile.

Our subsequent investigation revealed for the first time that *C. pneumoniae* uses its inclusion membrane protein Cpn0308 to interact with ACBD3, thereby recruiting PI4KB to the inclusion site for its replication. Both ACBD3 and PI4KB are critical for the development of *C. pneumoniae*. These findings were corroborated by knocking out ACBD3 in HeLa cells and inhibiting PI4KB using PIK93. In ACBD3-knockout HeLa cells, the growth and replication of *C. pneumoniae* were significantly reduced, along with decreased expression of both Cpn0308 and PI4KB. The aggregation of PI4KB near inclusion bodies was not observed. As demonstrated in Lyoo’s study (35), the delayed enterovirus replication in ACBD3-knockout HeLa cells was also observed, with no recruitment of PI4KB in infected ACBD3-knockout HeLa cells. Similarly, in our study, HeLa cells treated with PIK93, a specific PI4KB inhibitor, exhibited reduced replication of *C. pneumoniae* and decreased expression of Cpn0308. Although we did not observe any change in the expression of PI4KB following PIK93 treatment, this finding aligns with Li’s study on human parainfluenza virus type 3 (HPIV3) (43). The study reported that treating the HeLa cells with PIK93 for 1 h did not affect the expression or co-localization of PI4KB on inclusion bodies but significantly reduced HPIV3 replication. The reasons for these observations may include insufficient concentration and duration of PIK93 exposure, or the possibility that PIK93 might act on downstream molecules of PI4KB, thereby interfering with the replication of *C. pneumoniae*.

Li’s research demonstrated that PI4KB is capable of producing PI4P on HPIV3 inclusion bodies, which is essential for viral replication; inhibiting PI4KB can block the synthesis of PI4P (43). Analogous findings have been observed in enteroviruses 71 and 68 infections of HeLa cells, where suppression of PI4KB hinders both viral replication and PI4P production (29). Consequently, we examined the concentrations and localization of PI4P within *C. pneumoniae*-infected HeLa cells. Our results revealed that *C. pneumoniae* facilitated the co-localization of ACBD3 with PI4P, as well as Cpn0308 with PI4P, within inclusion membranes in infected wild-type HeLa cells. Given our discovery that Cpn0308 interacts with ACBD3, thereby recruiting PI4KB to inclusion membrane, and considering that PI4P in humans might be generated through the action of four phosphatidylinositol 4-kinases: PI4KIIa (PI4K2A), PI4KIIb (PI4K2B), PI4KIIIa (PI4KA), and PI4KIIIb (PI4KB) (44–46), we hypothesized that *C. pneumoniae* employs PI4KB to recruit PI4P to the inclusion membrane and facilitate PI4P synthesis. Subsequently, we treated Hela cells with PIK93, a specific PI4KB inhibitor, which resulted in decreased levels of PI4P (Fig. 6B) and reduced replication of *C. pneumoniae*. The results were consistent with those of Li’s study and the enterovirus research (43). Additionally, this result also clarified that PIK93 treatment does not impact the expression of PI4KB as described above; instead, it causes a decrease in the levels of its downstream molecule PI4P.

Moorhead’s group (33) demonstrated that during *C. pneumoniae* infection, host proteins OCRL1 (Oculocerebrorenal syndrome of Lowe protein 1) and PI4KIIα (phosphatidylinositol 4-kinase IIα, PI4K2A) were also recruited to chlamydial inclusions, promoting the inclusion membrane localization of PI4P and regulating its levels, which are essential for chlamydial development. They also showed that OCRL1 recruitment to inclusions is mediated by Rab GTPases, key regulators of membrane trafficking, which are known to be recruited to chlamydial inclusions in infected HeLa cells (33). The study suggested that these proteins may perform partially overlapping functions in the development of *C. trachomatis*. In contrast, our study found that PI4KB is recruited to the inclusion membrane by host protein ACBD3, which regulates PI4P levels. These findings raise questions: Is there a relationship between the Rab GTPases-OCRL1-PI4P pathway and the ACBD3-PI4KB-PI4P pathway in C. *pneumoniae* infection? Do they also perform overlapping functions in the development of *C. pneumoniae*? Since we identified C. *pneumoniae* utilized its inclusion membrane protein Cpn0308 to recruit ACBD3 to the inclusion and interact with it, whether Cpn0308 interacts with Rab GTPases remains to be determined, which might help us to find the relationship between these two pathways and their functions. As ACBD3 has several binding partners at the Golgi, including Giantin, Golgin160, PI4KB, and Rab33b GA (47–50), whether there exist other pathways by which ACBD3 acts with its partners for *Chlamydia* development also needs exploration. In addition, both our and Moorhead’s studies indicated that *Chlamydia* promotes the localization and synthesis of PI4P in the inclusion membrane to facilitate its development. How does PI4P affect the development of *Chlamydia*? Does it participate in the metabolic pathway in inclusion bodies? We are currently verifying these issues.

In conclusion, our research has revealed a pivotal mechanism by which *C. pneumoniae* employs its inclusion membrane protein Cpn0308 to engage in a targeted interaction with the host’s ACBD3 protein within the confines of the inclusion. This initial engagement sets off a cascade that recruits PI4KB to the site, ultimately stimulating the generation of PI4P—a crucial step that propels the growth and replication of the pathogen. The research underscores the critical importance of both ACBD3 and PI4KB in the replication process of *C. pneumoniae*, potentially attributing this to their intracellular localization. It is plausible that the pathways involving ACBD3, PI4KB, and PI4P are manipulated by Cpn0308 to support the pathogen’s lifecycle.

## MATERIALS AND METHODS

### Cells and *C. pneumoniae* culture

HeLa cells were cultured in Dulbecco’s modified Eagle’s medium (DMEM, Gibco) supplemented with 10% heated-inactivated fetal bovine serum (FBS, GEMINI), 1% l-glutamine, and 1% penicillin and streptomycin solution and at 37°C in an atmosphere of 5% CO_2_. *C. pneumoniae* were cultured in Dulbecco’s modified Eagle’s medium (DMEM, Gibco) supplemented with 10% heated-inactivated fetal bovine serum (FBS, GEMINI), 0.25% gentamicin, and 1% l-glutamine and at 35°C in a 5% CO_2_ humidified atmosphere.

### Antibodies, reagents, and kits

A mouse anti-Cpn0308 polyclonal antibody was prepared in our laboratory. Rabbit anti-ACBD3 antibody (Cat. 14096-1-AP) was purchased from Proteintech. Rabbit anti-PI4KB antibody (Cat. ab134756) was purchased from Abcam. Mouse anti-PI4KB antibody (Cat. 611816) was purchased from BD. Mouse anti-GAPDH antibody (Cat. AC002) was purchased from ABclonal. Rabbit anti-β-actin antibody (Cat. AF5003), HRP-labeled goat anti-rabbit IgG (H + L) (Cat. A0208), Alexa Fluor 488-labeled goat anti-mouse IgG (H + L) (Cat. A0428), Cy3-labeled goat anti-rabbit IgG (H + L) (Cat. A0516), Cy3-labeled goat anti-mouse IgG (H + L) (Cat. A0521), and Cell lysis buffer for Western and IP (Cat. P0013) were purchased from Beyotime Biotech Co. Mouse anti-PI4P antibody (Cat. z-p004) was purchased from Echelon Biosciences. FITC-labeled sheep anti-mouse IgM (Cat. bs-0368G-FITC) and PE-labeled sheep anti-mouse IgM (Cat. bs-0368G-PE) were purchased from Bioss. Universal avoidance antibody heavy chain and light chain secondary antibody (Cat. M210081) was purchased from Abmart.

Protein A/G PLUS-Agarose (Cat. SC-2003) was purchased from Santa Cruz. Taq DNA Polymerase (Cat. KGF2207- 6) was purchased from KGI Biologics. dNTP Mix (Cat. P032-02) and 2× Rapid Taq Master Mix (Cat. P222-01) were purchased from Vazyme. Taqman probes were synthesized by Sangon. EnGenTM Spy Cas9 NLS (20 µM) (M0646T) was purchased from NEB. Prestained Standard Protein Marker (Cat. 26616) and DMEM cell culture medium (Cat. 2810684) were purchased from ThermoFisher. 2.5% glutaraldehyde (Cat. G1102) was purchased from Sevier.

Endo-Free Plasmid Midi Kit (Cat. D6915-03) was purchased from Omega. Neon transfection system 10 μL kit (Cat. MPK1096) was purchased from Invitrogen. Lipo3000 transfection kit (Cat. L3000015) was purchased from ThermoFisher. Genomic DNA Small Volume Extraction Kit and BeyoECL Plus (Cat. P0018S) were purchased from Beyotime.

### Immunofluorescence staining and confocal microscopy

The cells were cultured on the 24-well glass slide. The medium was first removed, and the cells were washed three times with phosphate-buffered saline (PBS). They were then fixed with 4% paraformaldehyde for 30 min and subsequently washed three times with PBS. After permeabilization with 0.1% Triton X-100 in PBS for 10 min, washed three times with PBS and then blocked with 10% goat serum in PBS for 1 h at room temperature. The corresponding primary antibody was incubated for 2 h at room temperature and washed five times with PBS for 5 min, and the corresponding secondary antibody was incubated for 1 h at room temperature and washed five times with PBS for 5 min each time. DAPI was used for staining and sealing of nuclei. The results were observed and analyzed by laser confocal microscopy (Model: Olympus LX83-FV3000; Manufacturer: Olympus, Japan). Confocal images were acquired from ≥10 distinct fields per sample. Representative images shown in the manuscript were selected based on their consistency with observations across all examined fields.

### Construction of ACBD3 knockout HeLa cell lines using CRISPR/Cas9

According to the sequence of the human ACBD3 gene from NCBI (Gene ID: 64746), 20 bp sgRNA sequences in the first exon were designed and synthesized by Thermo Fisher. The sequences were as follows: sgRNA-A1: 5′-GCAGCAGCCGGAGATGGCGGCGG-3′, sgRNA-B1: 5′-GCTGAACGCAGAGCGACTCGAGG-3′, sgRNA-C1: 5′- CGACTCGAGGTGTCCGTCGACGG-3′. The Cas9 protein was co-incubated with the sgRNA for 10 min, transferred to cells using an electroporator, and placed in a CO_2_ incubator. The cells were placed in 96-well plates for monoclonal culture and screening. Finally, Western blotting and indirect immunofluorescence assay were used for detection.

### Immunoprecipitation and Western blotting

HeLa cells were washed three times with PBS and lysed on ice for 30 min using RIPA buffer supplemented with 1 mM PMSF, with gentle inversion every 5 min. The cell lysates were centrifuged at 13,000 × *g* for 10 min at 4°C. The supernatants were incubated with the indicated antibodies overnight at 4°C and then incubated with protein A/G magnetic beads for an additional 3 h at 4°C. After washing three times with pre-cooled PBS, the samples were boiled at 100°C for 5 min, ice-bathed for 5 min, subjected to SDS-PAGE gel electrophoresis, and transferred to a PVDF membrane. The membrane was blocked with 5% skim milk powder for 2 h at room temperature, followed by an overnight incubation at 4°C with the corresponding primary antibodies. The membrane was then washed five times with 0.1% PBS-T, incubated for 1 h with secondary antibodies, and washed five more times. Finally, chemiluminescence (ECL) was used for color development and analysis.

### Preparation of ACBD3-restored HeLa cells

The recombinant plasmid pcDNA3.1-ACBD3 was extracted using Endo-Free Plasmid Midi Kit. Five micrograms of recombinant plasmids were transfected into ACBD3 knockout HeLa cells using Lipofectamine 3000. The expression of ACBD3 was verified by indirect immunofluorescence and Western blotting.

### The transmission electron microscope (TEM) was used to observe the development of *Chlamydia* and structural changes in organelles

After infecting HeLa cells with *C. pneumoniae* for 12, 24, 36, and 48 h, the cells were harvested and fixed with glutaraldehyde, followed by overnight refrigeration at 4°C. The samples were washed three times with PBS (pH = 7.6) for 15 min each time, dehydrated through a gradient ethanol series (30%, 50%, 70%, 80%, 90%, and 100%; 15 min each), and transferred to acetone for 20 min. The samples were then embedded using acetone-resin mixtures in a stepwise manner: 3:1 ratio for 2 h, 1:1 ratio for 3 h, and 1:3 ratio for another 3 h. Subsequently, pure resin treatment overnight and thermal curing (5 h each at 35°C, 60°C, and 80°C) were conducted. Ultrathin sections (70–90 nm) were obtained using a Leica ultramicrotome, stained with uranyl acetate for 5 min and lead citrate for 5 min. After drying, the ultrastructure of each sample was observed by transmission electron microscope (Model: FEI TECNAI G2 12; Manufacturer: Thermo Fisher Scientific). TEM images were acquired from ≥10 distinct fields per sample. Representative images shown in the manuscript were selected based on their consistency with observations across all examined fields.

### Real-time fluorescence quantitative PCR (qPCR)

After infecting HeLa cells with *C. pneumoniae* for 24 h, total cellular DNA was extracted using a DNA Extraction kit. This DNA served as the template for real-time fluorescence quantitative PCR (qPCR). The recombinant plasmid PGBKT7-Cpn0308, prepared in our laboratory, was used as the positive control standard. The amplification procedure for the qPCR reaction was as follows: initial denaturation at 95°C for 5 min, followed by 40 cycles of denaturation at 95°C for 30 s and annealing/extension at 60°C for 60 s. Fluorescence signals were read at the annealing step of each cycle. Results interpretation: If the amplification curve is S-shaped and the Ct value is less than 30, it will be considered positive.

### Flow cytometry analysis

Intracellular PI4P levels were assessed using flow cytometry. At 24 h post-infection with *C. pneumoniae*, the cells were harvested, and an uninfected control group was prepared simultaneously. The collected cells were fixed with 4% paraformaldehyde for 20 min and permeabilized with 0.1% Triton X-100 for 10 min. After permeabilization, the cells were stained with an anti-PI4P antibody for 1 h at 4°C, washed two times with PBS, and then incubated with a PE-labeled goat anti-mouse IgM for 30 min at 4°C. After a final wash, the cells were resuspended in sheath fluid to facilitate flow cytometry analysis.

### Statistical analysis

All data presented in this study are derived from three independent experiments and are expressed as the mean ± SD. Figures were generated using GraphPad Prism software, while statistical analyses were performed using SPSS software. Comparisons between different groups were conducted using one-way analysis of variance (ANOVA), followed by Student–Newman–Keuls (SNK) tests or Student’s *t*-tests where appropriate. Significance levels are indicated as follows: NS (not significant, *P* > 0.05), * (0.01 < *P* < 0.05), ** (*P* < 0.01), *** (*P* < 0.001), and **** (*P* < 0.0001).

## Data Availability

The data sets used and/or analyzed during the current study are available from the corresponding author upon reasonable request.
